# Traditional Knowledge and Conservation Priorities of Eurasian Red Squirrel (*Sciurus vulgaris*) in Finland

**DOI:** 10.1002/ece3.71484

**Published:** 2025-05-26

**Authors:** T. Mustonen

**Affiliations:** ^1^ Snowchange Cooperative Lehtoi Finland

**Keywords:** boreal forests, Eurasian red squirrel, historic hunting, oral history, squirrel ecology, traditional knowledge

## Abstract

The Eurasian red squirrel (*Sciurus vulgaris*) has played an important role in Finnish traditional culture and livelihoods since pre‐historic times. This paper analyzes the current role, status, and trends of the red squirrel using both available scientific evidence and testimonies from oral historians who were immersed in Finland's boreal hunting societies during the mid‐1900s. The convergence of observations points to the decline of this iconic mammal of northern forests and increased migration to urban habitats. Red squirrels are not currently seen as being of great relevance to conservation efforts, despite their central role in spreading seeds and in boreal predator–prey food chains. If the animal is lost from Finland's remaining boreal timber forests, their absence may have consequences that are not yet understood. This article contains previously unavailable cultural knowledge of the Eurasian red squirrel, directly curated by knowledge holders. It presents an important nexus of different ways of knowing.

## Graphical abstract

Eurasian red squirrel ( *Sciurus vulgaris* ) has played an important role in Finnish traditional culture and livelihoods from pre‐historic times. Convergence of observations points to the case where the iconic mammal of the northern forests is in decline and migrating to the urban habitats, with a number of important consequences. If the animal is lost from the remaining boreal timber forest in Finland, their absence may have consequences that are not yet understood.

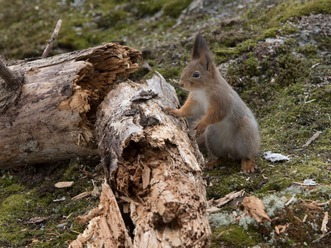

## 1 Introduction

On 10th July 2024, while driving through the village of Kiihtelysvaara in Eastern Finland, I spotted the carcass of a young squirrel on the highway. It had been run over by vehicles speeding down the hill south of the village center. A symbol of the boreal forest and the region, killed by modern traffic, lost in the ditch, abandoned, forgotten…

**FIGURE 1:**
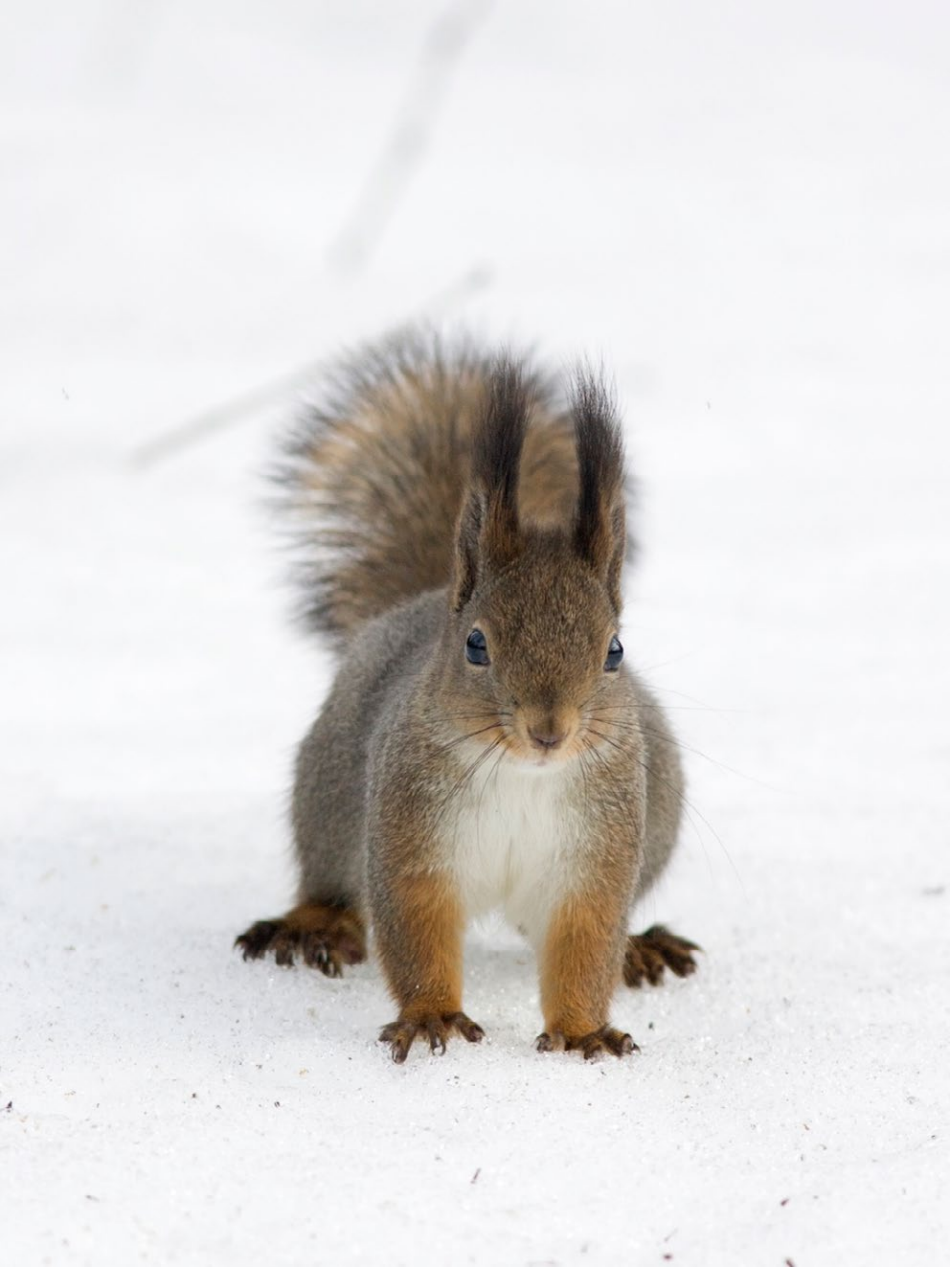
The digits of the squirrel need to be spread wide apart to enable it to travel on top of the deep boreal snow. Photo: Eero Murtomäki.

Having conducted work on community‐based observations of climate and ecological change, including knowledge of mammals in the Eurasian North, for almost 30 years, I have recently been pondering the role, status, and trends of one of the most iconic animals in Finland: the Eurasian red squirrel ( *Sciurus vulgaris* , see Figure 1).

This article aims to discuss both the recent understanding of the status and trends of the Eurasian red squirrel, as well as its role in Finland's cultural knowledge matrix. It includes snippets of embodied sensory and emotional responses to observing red squirrels that have emerged during this research effort. Lastly, it showcases images of the red squirrel, captured by Finland's longest‐serving nature photographer, Eero Murtomäki. These illustrate squirrels behavior and life in the boreal forest, their natural environment.

Hellstedt and Laaksonen (2022) indicated that the Eurasian red squirrel arrived in Finland 6000–8800 years ago. Red squirrels can live up to 12 years of age (Leppänen 2005). They typically weigh 200–400 g, with an average of 330 g, and usually grow between 18 and 24 cm in length. Hellstedt and Laaksonen (2022) reported that squirrel territories are usually found in boreal forest ecosystems and are between 3 and 30 ha in size, with an average, measured in Nuuksio National Park, of 28 ha (see also Leppänen 2005). Territories of 120 ha in size have been reported in Sweden. Steiner and Huettman (2023) wrote that all 280 different squirrel species found globally play an important role in ecosystems through, for example, seed dispersal and as bioindicators. Steiner and Huettman (2023) noted that squirrels' positive ecological impact is greatly undervalued.

The Eurasian red squirrel in Finland is considered to have two main color types, developed due to stronger association with either Norwegian spruce or Scots pine forest ecosystems and the need for camouflage (Hellstedt and Laaksonen 2022). In the northern parts of Finland where Scots pine dominates, squirrels are more red‐colored. In more southerly regions, where red squirrels live primarily in areas dominated by spruce, the animals' fur tends to be grayer and darker in the tail and ears. Leppänen (2005) reported that there are actually three color types, pointing to the existence of a brown‐tailed sub‐population. Lampio (1972) mentioned that, at an individual level, squirrels can express mixed color types combining all of the above, as well as albinism and melanism.

**FIGURE 2:**
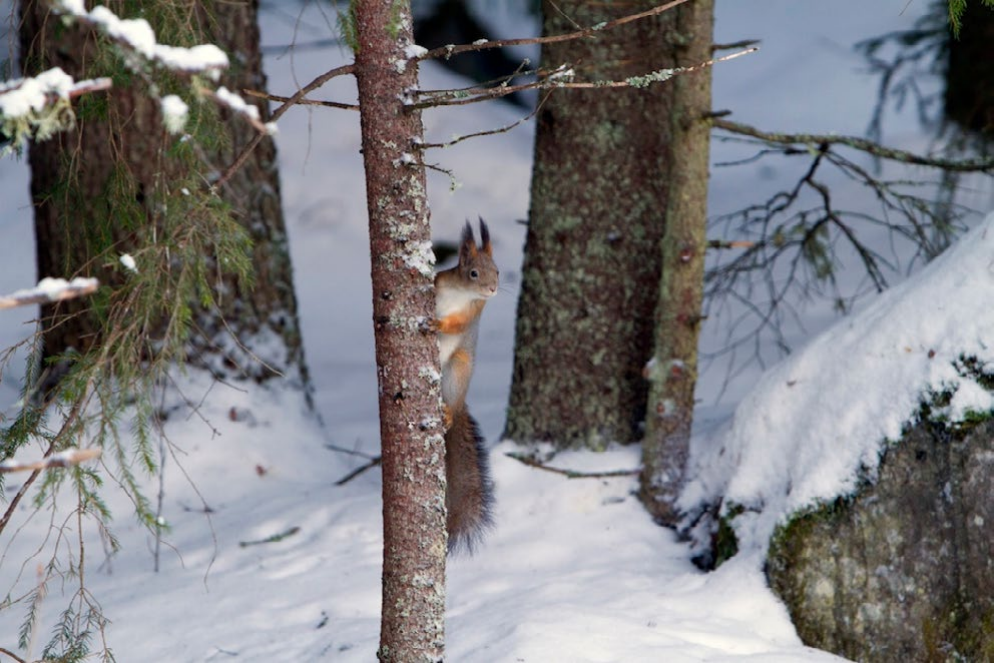
On a bad year of spruce cones, the food is scarce. Photo: Eero Murtomäki.

**FIGURE 3:**
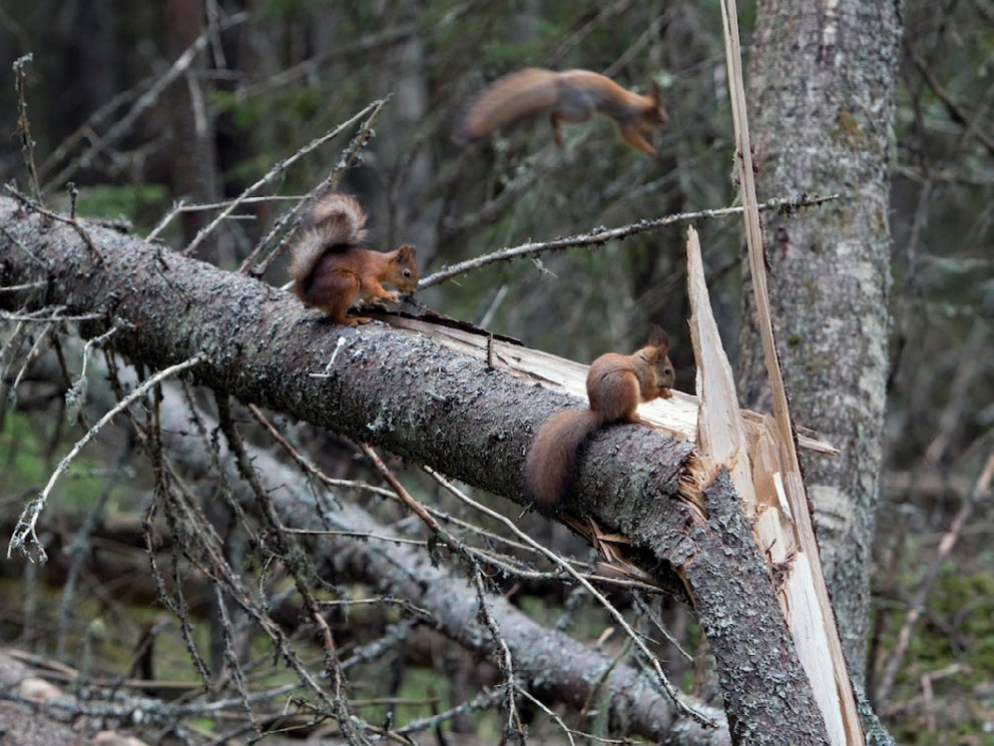
Two young squirrels and a mother. Photo: Eero Murtomäki.

**FIGURE 4:**
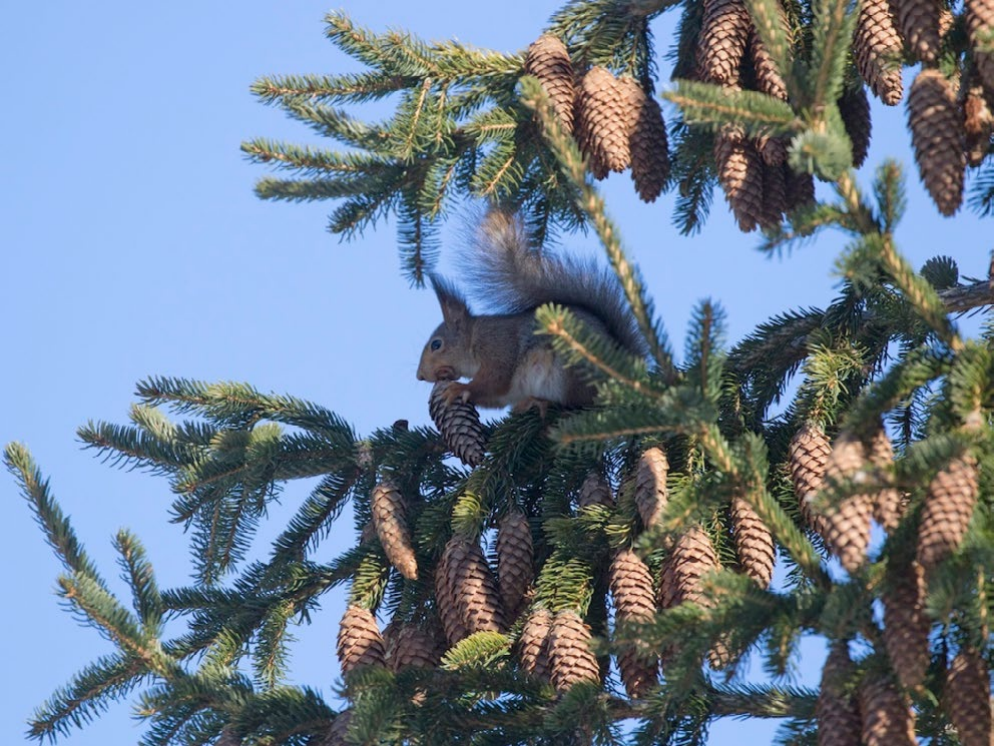
The stumps of overwintered cones can be used for food. Photo: Eero Murtomäki.

Shifts in the population of red squirrels can be dramatic. In years where cone production in the boreal forests is good, the mothers have 2–4 litters of 4–6 young squirrels (Figures 2 and 3). In years when cone production is low, mothers may only raise 1–3 offspring in an entire year. Lampio (1972) reported that spruce cone feeding areas may produce better survival conditions than pine‐dominated forests (Figure 4). In the past, coccidiosis (a parasitic disease) caused large natural mortality events.

Leppänen (2005) reported on a very interesting topic, that of squirrels undertaking large migrations of hundreds of kilometers towards a cardinal direction due to shortages of food and the development of high population densities. Lampio (1972) says that in 1819 in the United States a migratory group of approximately 450 million individuals spread across a 200‐km wide “front” was observed. Lampio (1972) provides us with a summary of known migrations in Finland:

- 1671—a large migration, followed by hunting families
- 1751, 1840, 1866, 1878, 1901, 1906—reports of migrations (no geolocation)
- 1916—migration on Lake Päijänne, central Finland
- 1923 and 1943—migration on Lake Kianta, eastern Finland

Squirrel populations in Finland collapsed at the end of the 1800s due to a combination of natural cycles and hunting pressure. In 1929, the species became fully protected in Finland. Red squirrels increased in number as a result. After the Second World War, recovering population numbers enabled hunters to harvest a large number of the animals each year (Hellstedt and Laaksonen 2022). Red squirrels were, at this time, the most lucrative catch for hunters in economic terms. The testimonies of oral historians who contributed to this article are drawn from this post‐WW2 era.

Red squirrel populations are perceived to have been declining again since the 1950s (Turkia et al. 2018). Seasonal squirrel distribution is also changing, with a growth in the populations found in urban areas during winter due to greater food availability (Turkia et al. 2020). Hellstedt and Laaksonen (2022) estimated the current population of red squirrels in Finland at 1 million individuals. The Bern Treaty of 1986 that enables conservation of wildlife came into force in Finland, enabling the legal protection of red squirrels nationally. It is the most important document to enable survival of the species internationally (Hellstedt and Laaksonen 2022).

Turkia et al. (2020) identified that conifer seeds are the red squirrel's main food resource in boreal forests. Its main predators are the pine marten ( *Martes martes* ) and birds of prey, especially the northern goshawk ( *Accipiter gentilis* ). The scientific literature points to the growth and decline of red squirrel numbers following fluctuations in cone crops over large spatial areas (Gurnell 1983; Andrén and Lemnell 1992; Turkia et al. 2020; Steiner and Huettman 2023).

The red squirrel is known to most Finnish people. We have had a long cultural relationship with the squirrel. Any assessment of this species' ecology, exact numbers, and shifting situation can be enhanced by the use of traditional knowledge observations (Selonen and Hanski 2015; Selonen et al. 2016; Turkia et al. 2020; Steiner and Huettman 2023).

In this article I ask: what kinds of cultural knowledge exist about the red squirrel in Finland? To answer this question, I draw on scientific data and three oral histories from bearers of traditional knowledge associated with the red squirrel: two hunters from eastern Finland and one from central western Finland. All were associated with the last major professional squirrel hunts of the 1940s and 1950s (Turkia et al. 2016). These three oral histories constitute the analytical center of the manuscript. The main aim of this approach is to offer knowledge from living memory that has not previously been available in the literature. Additionally, I will draw from other relevant oral history materials, as well as a range of scientific and historical publications. I will also draw on some of my own observations and experiences to highlight key points.

I include oral history snippets from the archives and literature to highlight the relationship and kinds of knowledge the “last squirrel hunters” in Finland had. The key knowledge holder, Väinö Viitapohja (2003), a hunter from the Western part of Finland, provides particularly important cultural‐ecological reflections on the reasons for the decline in red squirrel numbers in Finland's boreal forests. His testimony demonstrates how traditional knowledge and oral histories can play an important role in determining change and abundance over time in boreal ecosystems (Johnson et al. 2015).

Testimony like Väinö's is particularly precious because, though it is still permitted to harvest squirrels in Finland from November to mid‐winter (https://riista.fi/game/orava/, Turkia et al. 2016), the role and scope of the hunt have been diminished and cannot be compared with the professional harvests of the mid‐20th century (Turkia et al. 2016).

## 2 Literature Review of Cultural Knowledge of the Red Squirrel

The cultural importance of the red squirrel is reflected in Finnish traditional culture (Voionmaa 1947). The national author of Finland, Aleksis Kivi, raised the profile of the squirrel with a poem entitled Oravan laulu, which was included in his classic 1870 novel Seitsemän veljestä (Seven Brothers) (Hellstedt and Laaksonen 2022). In many Finno‐Ugric languages, such as Mordva, Komi, and Mari, a word for squirrel skin means a *kopek*, that is, the smallest unit of domestic Russian money. The words ora and orav can be traced from proto‐Uralic languages (Hellstedt and Laaksonen 2022).

Hellstedt and Laaksonen (2022) reported that first evidence (bone findings) of a European squirrel hunt can be traced to 40–45,000 years ago. According to Hellstedt and Laaksonen (2022), 2500 years ago the production of iron increased the need for a means of exchange between peoples. Salt and other goods were exchanged with southern trading partners—a connection which also increased the volume and scope of the squirrel pelt trade.

Squirrel skins enabled the evolution of endemic cultural exchange (Mustonen 2014) and monetary concepts in Finland. Saloheimo (1976), Hulden (2005), Leppänen (2005) and Hellstedt and Laaksonen (2022) summarized these concepts and equivalencies:

- *Tikkuri* = 10 squirrel skins
- Four *tikkuri* was a *kiihtely*s, 40 skins, equal to two *rihma*, 20 skins
- In 1000 ad in Finland one *tikkuri* could buy 12 chickens, 2 *kiihtely*s could buy 2 bulls
- One skin fetched 6 L of beer in the 1750s
- The term *arvokas* (“valuable”) originally referred to the dense, gray winter fur of a squirrel
- *Raha* = “money,” in Modern Finnish, originally meant “squirrel skin” (Lampio 1972)

In 1950, the price of one squirrel skin was 110 Finnish marks (approximately €4 in 2024; Hellstedt and Laaksonen 2022). Leppänen (2005) and Hellstedt and Laaksonen (2022) report recent hunting numbers in Table 1.

**TABLE 1:** Numbers of squirrels hunted in Finland between 1996 and 2020.

Year	Animals hunted annually
1996–2002	2400–6300
2003	3600
2015–2020	1700–7200

As alluded to by the term *arvokas*, red squirrels were Finland's most valuable fur‐bearing animals from pre‐historic times to the fur trade era (Voionmaa 1947). Voionmaa (1947) and Turunen (1981, 233) identify the squirrel hunt as an important historic livelihood activity for the Finns (see also Saloheimo 1976; Turkia et al. 2016). According to Turunen (1981) squirrels were hunted with bows and traps, called oravatakka and loukku. The earliest descriptions of squirrel hunts bear out his conclusions, as they include references to traps and the use of arrows which, as we will see below, persisted well into the historic era. The value of squirrels was so great that famous Karelian hunters from what is now far‐eastern Finland, such as Ignoi Vornanen, traveled across the country to participate in squirrel hunts (Voionmaa 1947).

As well as possessing great economic value, the red squirrel also held great spiritual and symbolic importance. Turunen (1981) writes that the squirrel was culturally recognized as a symbol for speed or small size and it is present in spells and magic (Laitila 2017; Turunen 1981, 233). Specialized language evolved around the squirrel. For example, the word Oravikuusi, used to describe a spruce tree where squirrels live, is further evidence of the animal's cultural importance (Turunen 1981, 233). Hulden (2005) mentions that a special symbol associated with the squirrel was inscribed on old rune staff calendars from the 1600s to mark the time of the squirrel hunt. Hellstedt and Laaksonen (2022) mention that squirrels visiting the yard of a home were interpreted as a dark portent. Records also exist for the belief that red squirrels spread leprosy, caused forest loss, and the loss of game birds. These beliefs may have been associated with the proliferation of farming in western Finland and its associated impacts (Haavio 1967).

People who still live in rural areas of Finland today carry living memories of the cultural role squirrels played in village life. As part of this research effort, Parviainen (2006) shared a story told to her as a young person:

… if a squirrel runs into the home yard where you are living, that means that someone close to you, a relative or a person you know will die …. The place where we're living is an open field space. The forest is not very close. It is rare to see squirrels there …. Once I saw a squirrel and it was so terrible. I was so afraid about this that something would actually happen. But luckily nothing happened at that time.

This article is an inquiry into the role of red squirrels in Finnish traditional knowledge, as perceived by living knowledge holders. Three individual, documented oral histories constitute the main materials. Additionally, I include materials from linguistic neighbors—Karelian and Sámi oral histories of the animal—given the red squirrel's prominence in the boreal forest communities of both Finland and Sweden. Two of the oral historians I worked with hail from Kiihtelysvaara, a community whose name indicates a long cultural history with red squirrels (Saloheimo 1976). Nirvi (1974, 604) describes how, before the village site had been cleared in the forest, someone shot a kiihtelys (40) of squirrels on the site. This feat gave the eventual settlement that grew up in its place its name, Kiihtelysvaara, which translates into English as: Forty Squirrel Skin Hill. As previously described, this placename refers to an endemic (Mustonen 2014) form of value calculation related to squirrel pelts that eastern Finnish villages used until the early 1900s.

Oral historians are able to describe other cultural elements of the relationship between residents of Kiihtelysvaara and the red squirrel, but most of these traditions are no longer practiced and have been functionally lost. Peltonen et al. (1977, 172) present visual evidence of six squirrel skins that were “crucified” and placed under the church floor boards in the 1930s. They theorize that this was an act of protective magic, implying that the squirrel continued to have symbolic significance in the community well into the 20th century.

By examining old trading records, Voionmaa (1947, 383) describes the importance of squirrel‐related language and concepts used in economics, such as gråverk (gray skin), boghaverk (tax skins), and kreles werk (Karelian skins). In his regional dictionary, Nirvi (1974, 1341, 1342) describes and documents the linguistic diversity associated with red squirrels in Kiihtelysvaara. Word‐concepts documented by Nirvi (1974) include:

- *Oravalatinki*: a bullet for a smaller gun used to shoot squirrels
- *Oravanhank*i: autumn snow that is able to carry the weight of a squirrel, associated with the cultural aphorism: “If there is a snow that carries a squirrel in the autumn, there will be a snow that carries a male horse in the spring”
- *Oravanikenet*: very cold weather conditions
- *Oravarokka*: soup made from squirrel meat and groats
- *Oravakoira*: a dog used in the squirrel hunt
- *Oravikkotatti*: a fungus (*Boletus versipellis*) associated with the squirrel

Frainer et al. (2020) point to the role linguistic, cultural, and ecological elements have in the wider complex of biodiversity. Evidence from Nirvi (1974) indicates the central role that the red squirrel has played in shaping the local dialect and socio‐ecological landscapes around Kiihtelysvaara. They demonstrate linguistic connections between squirrels and specialized tools (weapons), weather events and snow qualities, foods, and other species associated with the squirrel. The May lily ( *Maianthemum bifolium* ), for example, is locally called *Oravanmarja* or *Squirrel Berry*, and has become the word used to describe the forest type where squirrels can be found (Hellstedt and Laaksonen 2022). This localized linguistic evolution may be indicative of an older way of conceptualizing and dividing use areas in the boreal forest.

In western Finland, similar linguistic connections have been influenced by Swedish. For example, Murtomäki (2024) associates *pihkanokka* (sap nose, referring to spruce sap) with the Swedish concept of *kådnos* (sap nose in Swedish). Knowledge holder Vierikka (2002) from western Finland shares an oral history snippet concerning squirrel hunting during WW2:

Ultimately I had no desire whatsoever to take part in hunting, even though I had a lot of enthusiasm towards fishing. This is due to my father, who was really enthusiastic about hunting squirrels (oravastaa) during war‐time. They paid a little more for the skins during wartime, even though the bullets cost a lot. He forced me to climb trees up which I didn't want to go! Sometimes the squirrel would get stuck in a fork up in the tree after being shot and we young people were made to climb the tree to get them! We needed to kick the tree trunk and hit it with sticks and so on. Like I said earlier, this experience meant I had enough of hunting squirrels (oravametsällä). I had no interest in hunting after that!

In Vierikka's (2002) oral history snippet, there are two interesting concepts associated with squirrel hunting. The first is the local dialect verb *oravastaa*, which refers to hunting squirrels. The second is the spatial–temporal concept of *oravametsä* (squirrel forest), which implies a specific time and place for squirrel harvesting. Both indicate the central practical and conceptual role the squirrel and the squirrel hunt held in community life.

Voionmaa (1947) discusses the meaning of *oravametsä*, or *oravimetsä* (p. 123), as he refers to them. According to him, the concept was a very important one, used as a general spatial reference for hunting territories from the 1300s onwards. Squirrel hunting territories (*oravametsät*) described the seasonal land use and occupancy of Finnish hunters and were strongly linked with *erämaa* (Mustonen 2014): the larger hunting territories in which they nested. Later, the territories where squirrel hunting took place became the subject of the very first form of land taxation in Finland.

Squirrels also became a part of society, identity, and descent through their use in naming customs. Mikkonen and Paikkala (1993, 394) report that the surname Orava or Oravainen (squirrel or squirrel‐like) is associated with totemic origins in eastern Finland. According to them, it may have been assigned to particularly talented squirrel hunters. The earliest mention of this surname dates from 1365 and a letter issued by King Albrekt Meklenburgian concerning prohibitions against Karelian and Häme hunters from the east. The surname was more prominent in southern Karelia in the 1500s and in Häme in the 1400s (Mikkonen and Paikkala 1993).

The founder of the written Finnish language, Mikael Agricola, compiled a list of Finnish forest deities in the 1600s. In this account, he writes: “Nyrckes Oraut annoi Metzast” (“Nyyrikki gave the squirrels from the forest”). This implies that the squirrel had an associated saint, Nyyrikki, who has been linked with the Catholic Saints Georgius or Bartholomaeus (Murtomäki 2024). Haavio (1967, 77) mentions that some of the early mythological sources (from 1789) point to a Tyytikki, a female forest god who was the mother, the *Emu*, of squirrels:

Tyytikki Tapion neiti (Tyytikki, daughter of Tapio)

Metzän pijka pikkuruinen (Smallest of the forest maidens)

Aukase rahanen aitta (Open up your chest of riches, of squirrel skins)

Mun metälle mentyäni (When I go to hunt)

Luinen lukkosi murra (Break tour bony lock)

Mieluussa mehtolassa (In the forest realm)

Et Emäntä Ijenekkään (Perhaps you are not the Lady after all)

Jos et anna ajallani (If you don't allow me to get any game)

iijälläni ilmuutak (Despite my efforts) (Freely translated by author)

**FIGURE 5:**
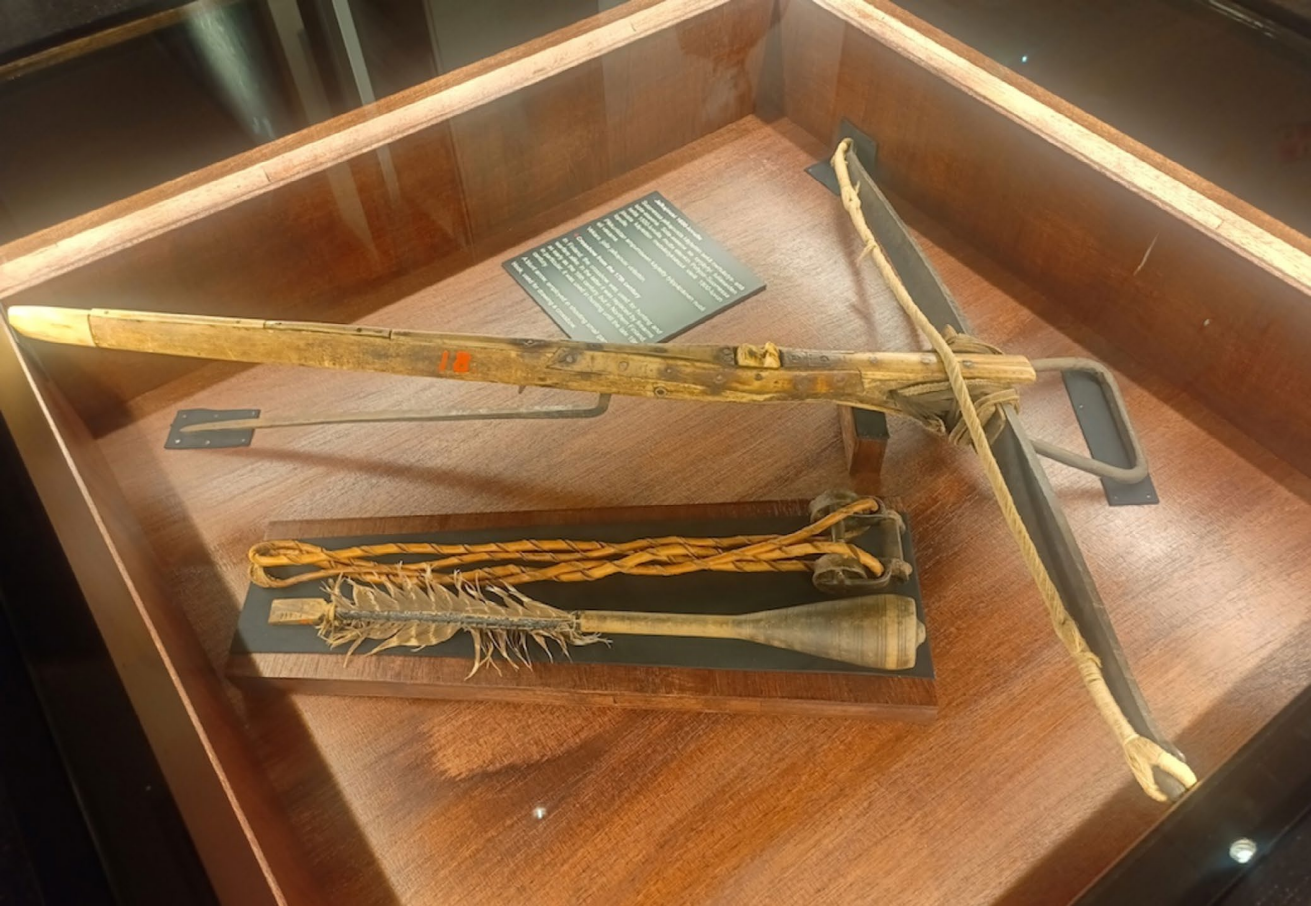
A squirrel hunting crossbow and arrows. Photo: Tero Mustonen.

In the 1442 Law of the Land issued by King Kristoffer of Sweden, it was decreed that fur bearing animals should not be hunted from Lent in the spring to All Saints Day in the autumn. Anyone violating these measures was given a sanction. The purpose of this decree was to ensure that fur bearing animals were able to reproduce in good numbers and to encourage the development of high quality pelts. Finland was, at that time, under Swedish rule. These decisions were also designed and decreed to inform hunting in the colony that would later become Finland. Björn (1991, 2006) says that in Karelia, eastern Finland, the squirrel hunt started at Kekri (All Saints Day). The squirrel was harvested mainly with blunt arrows that did not break the skin. These were fired using crossbows (Figure 5) which stayed in use well into the era of powder‐powered guns. Leppänen (2005) reports the ingenious invention and use of so‐called “whistling arrows.” The sound of these arrows caused the squirrel being hunted to freeze on the spot, making them an easier target.

Squirrels were eaten when they were caught, or skinned and taken to be eaten at home, especially in eastern Finland. In Lapland, squirrel meat was dried on the edge of the oven and crumbled into soups as a main course or as supplemental meat (Itkonen 1948). The Skolt Sámi Indigenous People fed squirrels to their dogs.

Huovinen (2006) shares an oral history from the White Sea Karelian community of Suomussalmi, Finland, which reveals the role squirrel hunting played in local seasonal cycles:

Each house had their own traplines called virkotie. During autumn they would store their catches in storage houses. During winter they would go, with horses or by other means, and collect these furs and catches. Mostly it was for birds. Squirrel was another very important animal in the hunting cycle, caught for its fur. It was mostly shot with a small rifle. In my time, when I became involved in hunting, it was with a shotgun. We mostly hunted squirrels in the early winter, on the autumn side, up until the trips to the marketplace in December.

Huovinen's testimony indicates the use of established household traplines, illustrating patterns that Mustonen (2024) describes as part of White Sea Karelian community land‐use and occupancy. Huovinen also describes the use of seasonal storage huts to store the winter's catch, a practice that sets White Sea Karelian land‐use apart from other Finnish squirrel hunting practices.

Voionmaa (1947) reported that the squirrel hunt was at its peak in the 1300s. Whilst these statistics do not necessarily paint a unified picture (i.e., there are large fluctuations in the numbers) they provide a window into early trading. In 1391, 400,000 skins were shipped to Tallinn, Estonia, by a single fur trader. In 1571, 4400 skins were purchased from a single western Finnish trader (Voionmaa 1947). Hulden (2005) pointed to a single year in the 1500s during which 25,000 squirrel pelts were traded. In 1605, in the southern Karelian trading town of Viipuri, 70,280 squirrel skins were sold (Voionmaa 1947).

In Finnish Karelia, historic squirrel hunting statistics dating to before the advent of large‐scale forestry in the Koitajoki River Catchment is available in Table 2.

**TABLE 2:** Numbers of squirrels hunted in Koitajoki, Eastern Finland between 1757 and 1926.

Year	Number of squirrels harvested
1757	200 Squirrels harvested from a single estate
1850	1000 Squirrels are sold annually to St. Petersburg
1862	2500 Squirrels from Koitajoki area
1865	1000 Squirrels from Koitajoki area
1868	6000 Squirrels from Koitajoki area
1872	2000 Squirrels from Koitajoki area
1926	3500 Squirrels from Koitajoki area

*Source:* Summarized from Björn (1991, 2006).

Concerning villages in the Koitajoki catchment that were ceded after the Second World War to the Soviet Union, Peltola (2019, 2021) reported that squirrel hunting remained a particularly important economic activity in the community of Ontrovaara.

In the Indigenous Sámi societies of northern Fennoscandia, the red squirrel has played an important role in the trapping economy, the tax system, as food, clothing, and more (Itkonen 1948). Itkonen (1948, 271) shares a record that describes Skolt Sámi men eating two to three squirrels in a meal and considering the meat to be very tasty. Itkonen (1948, 340) also describes *vuarrev‐sei'b‐kaulâs*, a neck‐liner made from squirrel skins worn by Skolt Sámi women. This piece of clothing was made by pushing several squirrel tails together on a piece of string. Itkonen speculates that *vuarrev‐sei'b‐kaulâs* is a traditional item of northern Eurasian clothing. Similar items are worn by the Sakha‐Yakut, Khanty, Mansi, and Evenk Peoples in the Amur region of Siberia. The Skolt Sámi paid their taxes using squirrel skins. Itkonen (1948, 53) reported that in 1517, one fox pelt was considered equal in trade value to 21 squirrel skins. Squirrels were also considered a food item in tax payments.

Bäer (2003), a Swedish Sámi leader and reindeer herder, shared an oral history concerning the way Sámi hunters in northern Sweden associated ecology, hunting practices, and place‐names as a means to transmit hunting knowledge down the generations:

There is a Sámi place name, Oarrinjarga, which can be translated as: Squirrel Cape. There are a lot of pine trees there and I think old Sámi associated that place with plenty of squirrels. It is strange theclues a hunter can get from the place‐names.

Kjellström and Tunón (2024, 157, 158) reported traditional Indigenous knowledge associated with squirrels from Sámi communities living further south in Sweden. They describe that when the Sámi harvested a squirrel, the bladder would be removed and dried. When eaten as part of a meal, the bladder could help cure urinary problems.

The red squirrel continues to be an important animal for Finnish people to this day. This is partially reflected in the connections experienced and reported by urbanized populations. These experiences are showcased in an unpublished archival text by Mustonen (not the author) (Mustonen 2023), who reports a story from western Finland, recorded in the 2000s:

I was a student in Tampere Polytechnic in the early 2000s. I lived close to the Kauppi forest area, where I used to walk my dog almost every day. One autumn day we were coming back from our walk and I saw an old man approaching large pine trees at the edge of the forest. As he moved closer to the large trees, he was making quiet sounds and there was a rustle in the trees. Squirrels started to descend the pine trunks towards the old man, as he was putting nuts in the small holes in the tree bark. We had stopped moving with the dog and just stood there observing the situation and the man started to tell me about his memories from the war. He explained that his troop had adopted an orphaned squirrel during the war and it lived with them in the dugout. He described how it was very difficult to hide anything from the little squirrel and it kept stealing all the best bits of food. The little animal gave them something different to think about and made the horror (of war) somehow easier to bear. He had been a young man during the war but never forgot the squirrel in the dugout. As an old man he had established a relationship with the squirrels in the Kauppi forest.

## 3 Review of Traditional Ecological Knowledge (TEK) From Three Oral Histories

In this section, I present materials from three oral historians: Pentti Hassinen and Eero Piipponen from the Kiihtelysvaara area in North Karelia, and Väinö Viitapohja from western Finland. Key observations from each oral history snippet are then discussed in relation to scientific contributions to the questions and topics the oral historians raise.

All three oral historians were involved, part‐time or, in the case of Viitapohja, full‐time, in post‐WW2 red squirrel hunting economies. The oral history excerpts presented here are drawn from a larger oral history corpus that explores the traditional knowledge held in Finnish villages.

The oral histories were documented with minidisc recorders, transcribed and analyzed, then returned to the knowledge holders for review and approval before final inclusion in the oral history archives held by the Snowchange Cooperative. For the purposes of this article, the primary materials have been translated into English.

At time of writing, two of the oral historians, Hassinen and Piipponen, have sadly passed on (Hassinen 2012; Piipponen 2012). Their observations have been used elsewhere (see, for example, Mustonen 2013) to determine ecosystem health, status and trends, and community histories. Viitapohja is now 89‐years‐old but still lives at home. He reviewed the oral history materials presented in this article and approved their use.

In recent years, the inclusion of traditional and cultural knowledge in ecosystem and species assessments has become more common in the Circumpolar Arctic and internationally (Burch 2010; Arctic Council 2013; Johnson et al. 2015). However, its application in Finland, especially in matters concerning rural communities, has been limited (Mustonen 2014). While Finnish research has begun to place greater emphasis on the role citizen science can play in knowledge production, applications of traditional knowledge remain rare outside the Sámi Home Area (Itkonen 1948; Johnson et al. 2015).

Frainer et al. (2020) assert the benefits of working in a biocultural framework that connects practical, linguistic, cultural, and ecological knowledge with ecosystem and species studies. This paper's exploration of the biocultural importance of the red squirrel provides a good example of how oral histories and traditional knowledge can enter into a mutually‐enriching dialog with established scientific literature, especially when approaching questions about species trends over long spans of time and in a broad range of spatial contexts. Similar work drawing on oral histories has taken place in Alaska (Burch 2012), helping to provide estimates concerning historic changes to the Western Arctic Caribou Herd from the 1850s onwards.

The use of Finnish oral histories and traditional, endemic knowledge (Mustonen 2014) can also complement citizen science approaches, as it does not create hierarchies between epistemologies. Rather, it enables a region, community, and individual oral historians to position their observations into a context‐dependent but broadly relevant corpus of materials, as we will see below.

## 4 Pentti Hassinen and Eero Piipponen—Oral Historians From Kiihtelysvaara in North Karelia

Pentti Hassinen was an oral historian, traditional knowledge holder, and hunter from Kiihtelysvaara, North Karelia. Together with Eero Piipponen, he was a cultural knowledge holder in a region where red squirrels have been central to the lifeways, local language, and biocultural relations for centuries (Peltonen 1977; Nirvi 1974). Both hunters, who are referred to mostly by their last names below, were respected knowledge holders in their community, holding an implied customary status that may have defined who had the capacity, perhaps even the rights, to convey communal histories (Burch 2010; Mustonen 2013) within and as a part of the local matrix of events. Piipponen, in particular, was considered to be a masterful oral historian with a very rich vocabulary, mastery of local dialect concepts, and storytelling skill.

Pentti Hassinen, also a locally revered oral historian, shared his oral histories as part of the larger Jukajoki River Restoration Project, which took place in the region in 2012 (Mustonen 2013). During this project, Hassinen recounted how he started to hunt squirrels:

When I was at school, I needed to skip out when the squirrel hunting started. I went hunting with my father. Squirrel skins were appreciated and valuable in those times. My father would skip working in the forestry sector, put the horse in the stable and we would go squirrel hunting, as it paid more money than forestry work. (Hassinen 2012, Oral History Tape)

Hassinen describes his role as a boy in the hunt:

My task was to knock on the trees so that father would see where the squirrel was and be able to shoot it. He mainly used a low‐powered rifle and a shotgun. I assumed the use of the rifle then, as a part of the hunt. This was done to make sure the skin stays intact. (Hassinen 2012, Oral History Tape)

Squirrel skins were mostly sought to earn additional money:

We took the skins to the market place in Joensuu (the regional town). Usually we could gather over a hundred skins to sell per‐season. There were so many lice, much more than a hundred! My sister and I had to find and remove them from our father every night after he skinned the squirrels! (Hassinen 2012, Oral History Tape)

Every part of the squirrel was used in Pentti's home. The meat was eaten:

Yes we ate it. Mother prepared stew from it. I liked the taste, especially when we put some pork with it. This added a bit more fat to the meal. So it was not so raw‐tasting. I'd say the steam in the stew made the sappy taste disappear. (Hassinen 2012, Oral History Tape)

Eero Piipponen is from the same community as Hassinen. He also participated in the oral history work undertaken during the Jukajoki River Restoration Project of the 2010s. Piipponen was considered a central repository of traditional knowledge in his village, Alavi‐Jukajärvi. He contributed to a number of oral history and local history projects (Mustonen 2013). He participated in squirrel hunts as a young man:

It was very dedicated and sharp work to find the squirrels. We had a dog that barked when he found the squirrel. He was so enthusiastic that he went to the forest to bark at them even outside the (hunting) season. The dog drove the squirrel to a certain tree, then you needed to succeed in shooting them there. (Piipponen 2012, Oral History Tape)

Piipponen and his family also sold the skins in the local market:

Yes, we took the skins to Joensuu for the November markets. We often sold the skins until January and each skin needed to be stamped by the local law officer to certify we had not poached them. (Piipponen 2012, Oral History Tape)

Turkia et al. (2016) corroborate Piipponen's observations, describing how regulations at the time (in 1940s) specified that pelts had to be tagged with a label indicating their province and municipality of origin.

As oral historians from Kiihtelysvaara, a place whose name originates from the squirrel hunt, Hassinen and Piipponen point to the central and complementary roles squirrel hunts played in the home economies of eastern Finland in the 1940s–1950s. Hassinen provides important descriptions of how the animals were used for food, a practice that was prominent in eastern Finland (Voionmaa 1947; Saloheimo 1976; Hulden 2005).

The number of animals the oral historians report taking and selling corresponds to reports from Turkia et al. (2016) and Hellstedt and Laaksonen (2022), who indicate squirrel numbers at that time had recovered sufficiently to enable a controlled hunt that played a meaningful economic role in rural communities.

These oral histories from Kiihtelysvaara must be understood in the context of a post‐WW2 economic decline and slump in the region (Saloheimo 1976; Björn 2006). This depression fostered greater reliance on wilderness economies such as hunting and fishing. Piipponen's discussion of the regional trading centre, Joensuu, and the controls enforced by the authorities indicates the significance and scope of the squirrel hunt at that moment in history.

## 5 Väinö Viitapohja—Hunter From Koskenperä, Virrat

Väinö Viitapohja worked as a squirrel hunter in his youth. He participated in oral history documentation activities regarding squirrels that took place in Tampere in the spring and summer of 2003. At the time he was interviewed, Viitapohja lived in the city of Tampere, Western Finland. However, his squirrel hunting areas were located in the village of Koskenperä, Virrat, approximately 100 km north of Tampere. Koskenperä is a village area dominated by boreal forests and peatlands. Viitapohja lived in Koskenperä in the early part of his life. He later made the return trip from Tampere to harvest squirrels professionally during the hunting season.

Viitapohja describes (Viitapohja 2003) the start of his hunting career:

In the autumn I received a pellet gun. I must have been around 10‐years‐old (approximately 1946). Then, as I got to be twelve, I had a dog and I was wandering in the forest and I was able to shoot and down a forest grouse. It did not die instantly but the dog was able to get it. I took the bird proudly to my uncle and he said—‘don't use the pellet gun, here is a real shotgun you can use!’ After that I became enthusiastic about hunting. (Viitapohja 2003, Oral History Tape)

Viitapohja's first squirrels followed soon after:

Squirrel hunting permits were given for the autumn. They issued 3‐week licenses for each month. I went out and hunted from dark‐to‐dark (morning to evening). We loaded the weapons alone in the dark, by candlelight. My days were spent out in the forest and I would come home with a backpack filled with squirrels. Mother would never let me in, as I had so many lice on me!

The dog was able to point to the squirrels. Sometimes they were in such big trees that I needed to climb up them, as you could not shake them. I had also acquired a small rifle from my uncle and I had to use that in the younger, planted forests. Shotguns should be used among the larger trees. Sometimes the squirrels would have such a secure grip on a larger tree with their fangs that when they died that it was impossible to retrieve them. (Viitapohja 2003, Oral History Tape)

Leppänen (2005) and Turkia et al. (2016) confirm that dogs were often used in the hunt. This required a high level of training, enabling hunters and dogs to work together. Viitapohja stressed the role of the dog in the harvest, describing his average hunting successes:

Usually I was able to get approximately 40 squirrels a‐day. A mean number, perhaps 30–40 a‐day. I had a really good dog and back then there used to be a lot of squirrels (in the 1940s). In the first year (after hunting) I had to go to the sauna and take all my clothes off due to the lice. I skinned the animals on a wooden board. I made those boards and you pulled the skin backwards to dry on the boards. They needed to dry there in the sauna for some days. (Viitapohja 2003, Oral History Tape)

Viitapohja, who was from western Finland, gave the meat of the animals to a couple who were originally from eastern Finland:

There was this old couple, their name was Kainulainen. I had nothing to do with the bodies of the squirrels myself, but they were asking for the meat and bodies. They asked me to gut them. I remember one winter, all‐in‐all I caught 600 squirrels and I took each one of them to their place for food. They kept each carcass in vinegar water first and then added salt. They ate every single one of those squirrels. They allowed me to taste the meat. It did not taste like sap. Perhaps the vinegar took that taste away. Otherwise it would have tasted so much like sap. (Viitapohja 2003, Oral History Tape)

In this oral history, we can see a marked difference in the ways western and eastern Finnish communities utilized squirrel meat. Viitapohja described a couple who lived in the area but originated in Karelia and who liked the meat, much like the Karelian communities described in Piipponen and Hassinen's eastern Finnish oral histories. The overall number of animals Väinö reports catching—in the range of 600 individuals—is an important spatial–temporal indicator. We will return to this insight below.

When, as a young man, Viitapohja took skins to Tampere, the local town, to be sold, he ran into some unexpected trouble with local law enforcement (Turkia et al. 2016):

I took my first trip out to Tampere to sell the skins. I agreed on a time to get there and I got to the railway station with my 600 skins in a cardboard box, all dried up. The buyer got there and wanted to see them, to open the boxes and check they were ok and of good quality. We were almost taken to jail when the local policeman showed up there and said that it is illegal to sell (furs) at the station. We had to scurry. He drove us out! (Viitapohja 2003, Oral History Tape)

Moving on to Viitapohja's observations and knowledge of squirrels in their natural habitat, he described the good hunting areas as follows:

It was really about the good cone areas. I knew what types of forest were in different parts. That was the key. I learned to identify good feeding areas, especially in young, maturing forest areas where there were a lot of cones. Also their (the squirrels') wintering areas, which tended to be these old spruce‐dominated forests. They were their wintering areas and they started to congregate there for the winter. (Viitapohja 2003, Oral History Tape)

This observation is in‐line with established scientific understandings of squirrel behavior, feeding patterns, and winter use areas (Turkia et al. 2020; Hellstedt and Laaksonen 2022). During the oral history work, Viitapohja reflected on the drivers of change in squirrel abundance from his hunting years to the time he was interviewed (early 2000s):

In the same autumn we rarely went to the same locations for the hunt. I had such a large territory^1^ to use. If the dog had been silent for 15‐min, we could move on. It was not a good spot. There were so many squirrels that it was not a problem to move on.

Nowadays, in the areas where I hunt moose the squirrels are gone. It is very rare to find squirrels there. One of the reasons for the change is the loss of tree‐hanging lichen in the trees. For some reason it started to disappear. Something happened with that, and it coincided with the disappearance of the squirrels. Also, the proliferation of the clear‐cuts took away the squirrels' feeding grounds. Areas where we had a lot of squirrels were lost completely and the animals migrated to other regions. I stopped squirrel hunting towards the end of the 1950s and early 1960s when other jobs became available. (Viitapohja 2003, Oral History Tape)

In this oral history segment, Viitapohja points to several important trends in the status and health of nature. When he was hunting squirrels in the decades following WW2 he was able to use a large area for the harvesting. He points out that the squirrels were plentiful at that time, affording him the luxury of “moving on” if a certain location did not have any squirrels. If we take Viitapohja peak annual squirrel take of 600 animals and divide this by the area of his hunting territory (10,000 ha), this implies that each squirrel harvested was using approximately 16.6 ha. Hellstedt and Laaksonen (2022) point to modern research that indicates that the average squirrel territory size today is approximately 28.8 ha per animal (see also Leppänen 2005).

The variance between these figures may reflect the existence of more intact habitats in Virrat in the 1940s and 1950s, meaning each squirrel needed less space to meet its needs, as well as the rebound in squirrel populations as a result of the animal being legally protected from the 1920s.

Scientific investigations into the reduction in squirrel numbers in Finland support Viitapohja's observations. Turkia et al. (2018) confirm the overall decline of red squirrels in Finland and in the wider region. Selonen et al. (2010, 2018) also observed a reduction in population numbers and assessed the reasons for the decline between 1950 and 2005 (see also Dylewski et al. 2016). Leppänen (2005) mentions the mass migrations squirrels have been observed to undertake but, when asked, Viitapohja said he had not seen these large migrations himself.

Selonen et al. (2010) determined that squirrels are relatively rare in the diets of the avian predators, with the exception of goshawks. Tornberg and Colpaert (2001) also identify goshawk as one of the predators of red squirrels in forested areas.

Selonen et al. (2010) confirm Väinö's observation that squirrel numbers declined between 1950 and 2005.

Selonen et al. (2016) indicate that predation is the main natural driver of declines in squirrel numbers in the wintertime (see also Selonen et al. 2018). The loss of habitat may exacerbate these natural drivers, leading to a cumulative impact on the species. This evidence supports Viitapohja's observations about the loss of feeding areas and wintering sites, and is in line with the findings of a large study conducted by Jokimäki et al. (2017), which detected a link between squirrel populations, increases in available food sources in urban areas, and loss of habitat in boreal forests. The authors found that while red squirrel populations are declining in Finnish forests, they are increasing in urban habitats.

**FIGURE 6:**
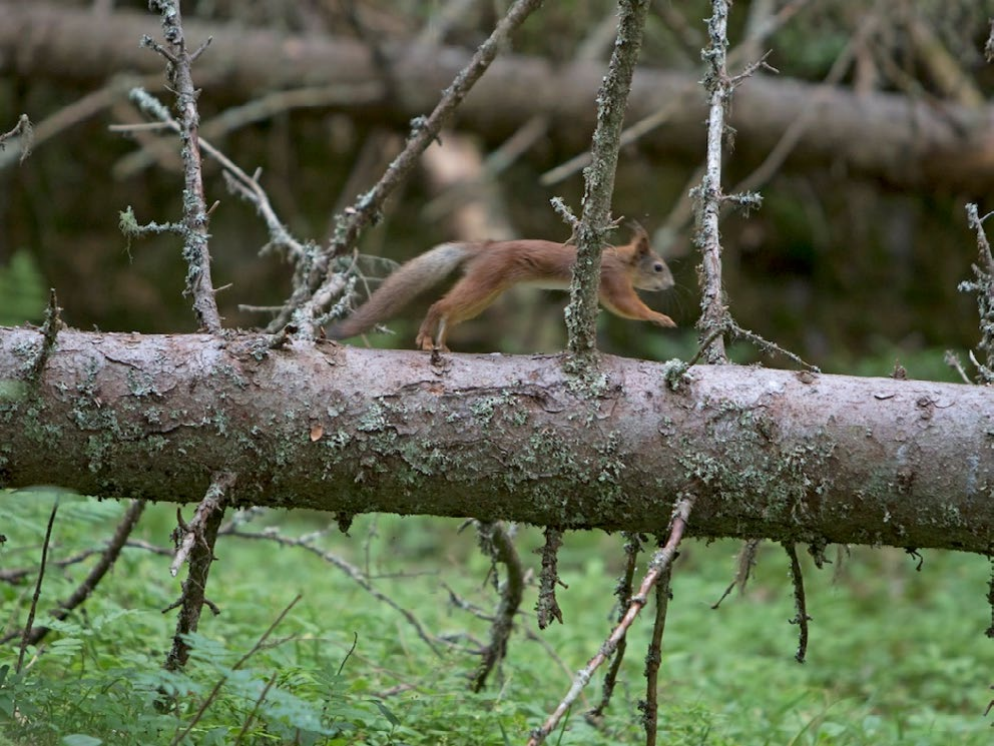
Young squirrels remain in their home forest for the whole summer. Photo: Eero Murtomäki.

Hämäläinen et al. (2019) confirm the squirrels' shift to urban habitats and altered ecological landscapes in their telemetry‐based study. They found a marked difference in the dispersal distances of younger squirrels (Figure 6) in urban areas compared to rural areas, indicating that dispersal rates in rural areas are approximately twice that of urban areas. Hämäläinen et al. (2019) detected change using land‐use classification of young forest (including clearcuts), birch‐dominated forest, pine‐dominated forest, spruce‐dominated forest, built landscapes (including buildings and roads), fields, and waterbodies.

Turkia et al. (2020) used snow track data from Finland spanning more than 29 years to argue that the main drivers affecting squirrel abundance are the availability and abundance of Norwegian spruce ( *Picea abies* ) cones and predation by pine marten ( *Martes martes* ) and the northern goshawk ( *Accipiter gentilis* ), probably due to shared habitat preferences. Turkia et al. (2020) stress that red squirrel population numbers are synchronized over remarkably large distances, a scale of hundreds of kilometers, and that this synchrony is mainly driven by a similarly spatially autocorrelated spruce cone crop. Viitapohja's use of large areas in the hunts of his youth provides corresponding observations of loss over large spatial areas and over time.

Continuing hunting into the 1990s and 2000s, Viitapohja observed a reduction in squirrel numbers in the locations where he hunted in Koskenperä, Virrat, in the 1940s and 1950s. Väinö attributes the decline in squirrel numbers to two major drivers. The first is the loss of tree‐hanging lichen (*usnea sp and bryoria sp*). These lichens were used by the squirrels to insulate their winter dreys.

Ympäristö (2025) as well as Hellstedt and Laaksonen (2022) indicate that, from the 1970s onwards, acid rain was a major driver in the decline of tree‐hanging lichens in Finnish forests. The acid rains were brought to Finland by atmospheric currents from Central Europe and Germany, where industrial factories released sulfur into the air via their smokestacks. Hellstedt and Laaksonen (2022) state that this has also affected Central European squirrel populations.

The second important driver of the reduction of squirrel numbers that Viitapohja points to is the intensification of forest management in Finland after WW2, and in particular from the 1960s onwards. He specifically identifies the clear‐cutting of boreal forests as a problem, saying that clear‐cuts have led to the destruction of important red squirrel feeding areas.

Hämäläinen et al. (2019) report that red squirrels occasionally move within clearcuts via the few trees left in the area. They found that these areas have very restricted seed‐bearing ability (see also Wauters et al. 2008). These findings support Viitapohja's observations about the impact of clearcuts on squirrel movements and feeding grounds.

Viitapohja offers further observations of how forested lands have changed:

Blueberry and lingonberry areas were much more plentiful in the past. We can see an expansion of lingonberry in clearcut areas, but otherwise there have been losses. We also had Arctic cloudberry, but it was lost in many areas when they started to drain the peatlands from the 1950s onwards. Especially in the larger peatlands. Also, forest roads have proliferated. In the past you could walk for kilometers without crossing any roads, but now there is always a forest road where you end up. I could navigate using the sun and the trees – which way the branches are growing in trees—and also the winds. On pine trees the branches tended to grow on the southern side. We could tell the time by looking at the sun.

**FIGURE 7:**
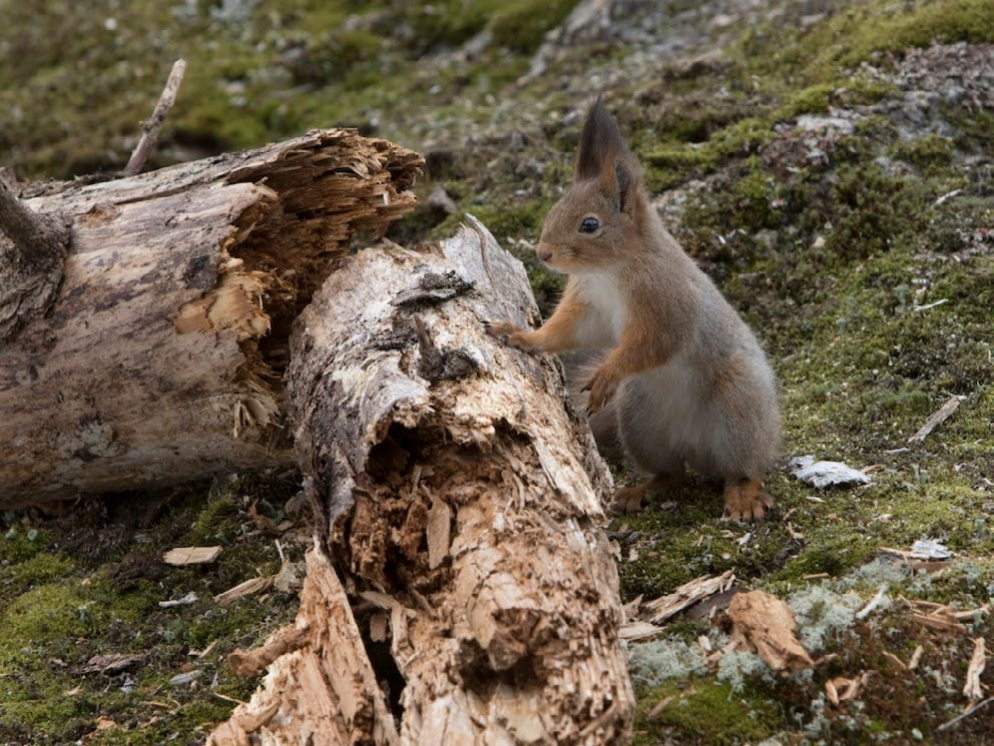
Maggots can be found living in forest deadwood. Photo: Eero Murtomäki.

**FIGURE 8:**
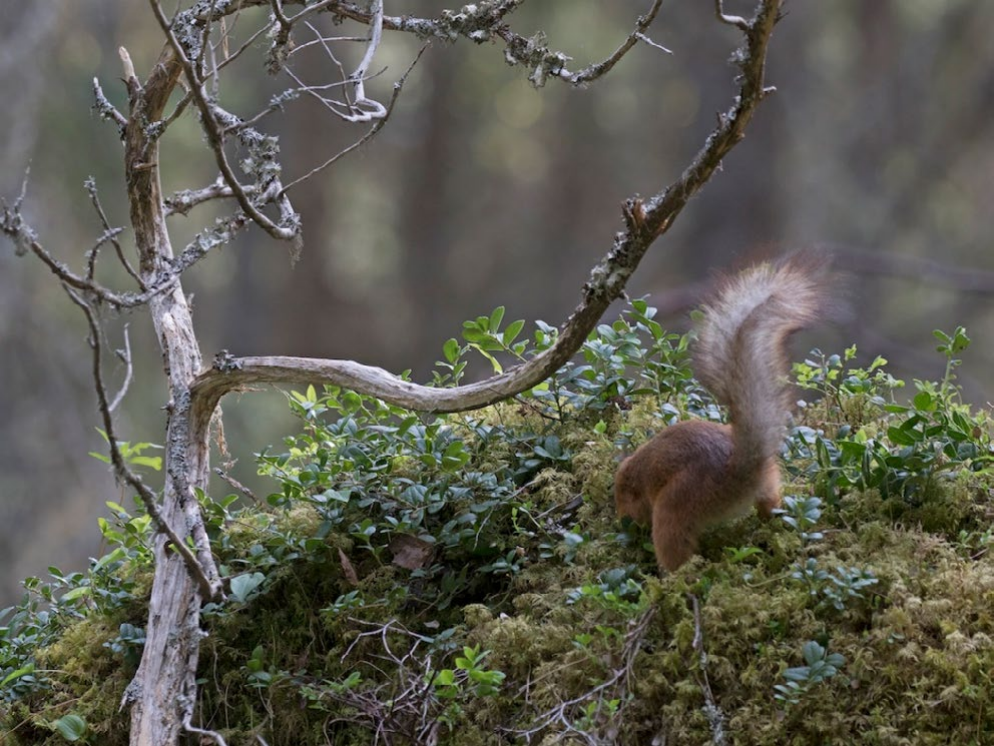
Squirrels maintain a large number of hidden food stores in the boreal forest. Photo: Eero Murtomäki.

Interviewed in the early‐2000s, Väinö reported large‐scale and significant changes in Finnish ecosystems. These natural losses had corresponding cultural and socio‐economic impacts. Traditional activities like berry‐picking, especially of Arctic cloudberry, had been affected by the loss of peatlands and clear‐cutting of primary forests. Developed in his youth, Viitapohja's ability to navigate using the natural signs found in primary forests (Figure 7) was also lost. Not through a loss of Viitapohja's own knowledge but through the loss of the ecosystems that provided these natural signs (Figure 8).

Viitapohja knew how to predict weather from natural signs. These included the behavior of insect‐eating birds like the black woodpecker, who would fly towards the ground when low pressure arrived, as well as air movements and certain clouds. For example, Karmutuuli, an extremely strong wind, was associated with the prediction of seasonal weather. Väinö remembers that in his childhood he was aware of the existence of noita: spiritual people who could inflict harm or find out about hidden things:

My uncle went to a noita, the old lady from Perälä. He wanted to find out where a lost cow was. The lady told him the exact location of the animal: next to a big rock, lying on its back, dead. My uncle said that he walked to the location and there the animal was. In our region we had two noita. People would go to them in secret. They were ashamed to go. (Viitapohja 2003, Oral History Tape)

## 6 Comparison With Other Sources

There is a long‐standing relationship between the Eurasian red squirrel ( *Sciurus vulgaris* ) and the cultures living in the territory of what is now the state of Finland. The animal held a central role in the hunting societies of the past. In this article I have drawn on interviews with three oral historians from eastern Finland and western Finland, all of whom participated in the large commercial squirrel hunts of the 1940s and 1950s.

It has often been claimed that the status and trends of red squirrel populations in Finnish ecosystems are little known (Hellstedt and Laaksonen 2022). The uses of squirrel skins, meat, and other parts, as well as the hunting complexes involving the animal, have been reported in literature (Voionmaa 1947; Hulden 2005), but authentic descriptions of squirrels by hunters themselves have remained scarce.

This research paper presents a number of important findings. Oral historians Piipponen and Hassinen from Kiihtelysvaara in eastern Finland describe the value, process, and specific elements of their hunts. They share that the partnership between a good hunting dog and a skilled hunter was essential to success in squirrel hunting. They also say that squirrel meat was seen as a valuable additional resource in the aftermath of WW2 and subsequent scarcity. Väinö Viitapohja, who hunted in western Finland, recounts a very different approach to the uses of squirrel meat as food, highlighting some geographical differences in the food security strategies and eating cultures contained within Finland.

Of the three oral historians involved in the work, Viitapohja provides us with the clearest reflections concerning changes in animal numbers and biogeographical environments over time. During his early life, he worked as a professional hunter in Virrat, western Finland. Later, he continued to visit and harvest moose in the same locations where he had once hunted squirrels, enabling him to provide vital temporal detail through his observations.

We hear from Viitapohja that his permitted use spanned a territory in the range of 10,000 ha and, in peak years, he was able to harvest up to 600 animals from this area per annum. This indicates that squirrels at that time were inhabiting territories of approximately 16 ha in size, which is feasible when compared with findings reported in scientific literature. That said, we must remember that a hunting team of one man and his dog would not have been capable of systematically harvesting every squirrel across such a large range. The numbers reported by Viitapohja indicate only those animals caught, and not escapees and unaffected individuals.

Viitapohja makes several ecological observations about the reduction in squirrel numbers over time caused, in his view, by factors including the loss of tree‐hanging lichen and clear‐cutting of trees in feeding areas. Selonen et al. (2010) and Turkia et al. (2018) point to scientific data that aligns with Viitapohja's traditional knowledge observations, indicating that red squirrel populations are in decline in most parts of rural Finland. Habitat loss caused by forestry and a switch in squirrel preferences towards urban environments are two of the drivers cited.

Turkia et al. (2020) do, however, caution that shifts in squirrel populations are very dynamic and connected with fluctuations in the seed production of coniferous trees over large areas of up to 655 km in radius. According to the authors, good cone years usually occur every 10–12 years and may cause the squirrel population to swell by up to 10 times. In times and areas of more dense squirrel inhabitation, weaker females are pushed into and reproduce in the margins of a territory (Hellstedt and Laaksonen 2022).

Habitat loss interacts with natural fluctuations in food sources and squirrel numbers by removing food sources from the landscape, affecting the capacity of forest areas to produce cones. When trees are cut too early, direct destruction of the dens of young animals, storage areas, and shelter may also occur. Turkia et al. (2018) report that squirrel populations are also affected negatively by climate change, especially warming winter temperatures. In comparison with these factors, predation may play only a small role.

Working with traditional knowledge holders like Piipponen, Hassinen and Viitapohja provides science and monitoring efforts with the opportunity to make important new discoveries, with insights and descriptions of bygone eras, overcoming the dangers of “shifting baseline syndrome”. The commercial harvesting of red squirrels as it was practiced in the 1940s and 1950s is unlikely to happen again. Along with these hunts other cultural roles played by squirrels have also disappeared, such as their prominence as a supplementary food source, as in the case of the Kainulainen family presented in Viitapohja's oral history.

It is also possible to appreciate the oral historians' voices as cultural texts which provide unparalleled insights into the relationship between red squirrels and Finnish forest villages in a critically important historic era. In Viitapohja's oral history, we also learn how the landscapes were monitored over time, how many animals were taken in a licensed hunting territory, and how these areas have changed over time. In general, the loss of habitat and loss of squirrels from their former ranges in Virrat seems to correspond with recent scientific investigations into the changes in red squirrel populations in Finland. This points to the important, currently underutilized role of traditional knowledge in assessing long‐term ecosystem change in Finland.

## 7 Reflections

During the summer of 2024, I observed a number of young squirrels that had lost their lives on the local road in Kiihtelysvaara, eastern Finland. Concerned about the disregard and disrespect these losses seemed to indicate, I embarked on a new path: one exploring Finnish relationships with the Eurasian red squirrel ( *Sciurus vulgaris* ) through both traditional knowledge and science.

In cultural terms, the squirrel is first mentioned in mythic times, as part of the older religion of the forest Finns recorded by Agricola (Haavio 1967). Squirrels were associated with certain deities and powers including the “mother spirit” of the animal. Finnish place‐names, despite the erosion of history, point to a totemic link between squirrels and people (Voionmaa 1947).

Itkonen (1948) and Kjellström and Tunón (2024) described how the Sámi people still preserve Indigenous uses of the animal in their culture. Well‐documented linguistic elements indicate that the central role of squirrels across the Finno‐Ugric realm is old indeed. Due to the central role of the fur trade in regional history, the old people associated words for money, calculations, and value with squirrels, indicating their central economic importance as one facet in a wider matrix of cultural values.

Entering into the modern era, in the 2000s, I was able to work with three oral historians to learn about the last great hunting complex associated with red squirrels in Finland: the commercial hunts of the 1940s to 1950s. In their own unique ways, each of these oral historians revealed that hunters greatly valued the squirrels, and that, in the eastern parts of the country, their meat was an important element of this relationship in addition to the skins. Väinö Viitapohja demonstrated concern over the loss of habitat, forests, and squirrel numbers through his decadal presence in his former squirrel hunting territories. This concern can be interpreted as revealing that hunters cared greatly about the animals they harvested.

When considered in relation to this historic context of care, value, and meticulous use, the young squirrels lying dead on the side of the road in Kiihtelysvaara appear as they are: indicators of a breakdown in the relationship between the squirrels and the people who share their territories. Today the animals are shifting to new urban habitats, benefitting from the food sources and “new” cityscape ranges created by domestic bird feeding and other sources of easy forage. The city's gain is the forest's loss. Leppänen (2005) and Steiner and Huettman (2023) point to the important role of the red squirrel in boreal forest ecosystems. These animals spread seeds and play a central role in boreal predator–prey food chains. If the animal is lost from Finland's remaining boreal timber forests their absence may have consequences we do not yet understand. Sadly, this appears to be the direction of travel. Turkia et al. (2018) confirm that over the past 29 years the red squirrel has declined in most parts of Finland. Sharing lessons from the Alaskan boreal, Steiner and Huettman (2023) highlight evidence of squirrel conservation needs and sound the alarm that, despite their importance, squirrels are not seen as being of great relevance for conservation efforts.

Those skeptical of the motivations of hunting societies may wish to conclude that the nature of relations between hunters and their prey (squirrels in this case) is strictly utilitarian; that the hunters were driven primarily by the financial value of squirrel pelts. Following Haavio (1967) and other cultural texts, as well as oral histories and my own life‐long embodied sensory and emotional experiences of observing red squirrels in the Finnish boreal, I argue that this utilitarian explanation is incomplete and ethnocentric. Yes, squirrels were harvested in the Finnish boreal over the centuries for their skins, meat, and tails. However, squirrels' material gifts formed just part of a larger matrix of endemic cultural values held by boreal communities, whose relationship with the squirrel was deep and more complex. Today, the European red squirrel comes to us as a messenger carrying a warning: our forests are in trouble.

During the writing of this article in July 2024, a young adult squirrel entered the main room of my home and stayed with me for 3 days. During this time, I discussed the purpose and scope of this inquiry with the animal and explained the need for us to revitalize our older relationship once more. He left the house soon after our discussion came to an end.

## Author Contributions

**T. Mustonen:** conceptualization (lead), data curation (lead), formal analysis (lead), investigation (lead), methodology (lead), resources (lead), supervision (lead), validation (lead), writing – original draft (lead), writing – review and editing (lead).

## Conflicts of Interest

The author declares no conflicts of interest.

## Data Availability

Data sharing not applicable to this article as no datasets were generated or analyzed during the current study. The quoted archival oral histories are publicly available in Finnish at the Snowchange Oral History Archives in Tohmajärvi, Finland.

## Endnotes

^1^
During the oral history work Viitapohja describes that the approximate use area for his hunting was around 10,000 ha. Most of these were state lands with a mix of some private lands. Viitapohja (2003) refers to the ease of the hunt. At that time there were no modern hunting associations who managed these use areas.
